# US Antibiotic Importation and Supply Chain Vulnerabilities

**DOI:** 10.1001/jamahealthforum.2025.3871

**Published:** 2025-10-03

**Authors:** Mariana P. Socal, Yunxiang Sun, Jeromie M. Ballreich, Jennifer Dailey Lambert, Tinglong Dai, Maqbool Dada

**Affiliations:** 1 Department of Health Policy and Management, Johns Hopkins Bloomberg School of Public Health, Baltimore, Maryland; 2 Johns Hopkins Carey Business School, Baltimore, Maryland; 3 Johns Hopkins Applied Physics Laboratory, Laurel, Maryland; 4 Johns Hopkins School of Nursing, Baltimore, Maryland; 5 Department of Anesthesiology and Critical Care Medicine, Johns Hopkins School of Medicine, Baltimore, Maryland

## Abstract

**Importance:**

The US has faced persistent antibiotic shortages over the past decade, compromising patient care, public health, and national security. Understanding the global sources of US antibiotic imports is critical to inform policies to improve supply chain resilience.

**Objective:**

To identify the global sources of US antibiotic imports, focusing on finished dosage forms (FDFs) and active pharmaceutical ingredients (APIs) between 1992 and 2024.

**Design and Setting:**

This cross-sectional study of US antibiotic importation records used data from USA Trade Online from January 1992 to July 2024. Data included import volumes, costs, and the originating country.

**Main Outcomes and Measures:**

Trends in annual import volumes for antibiotic FDFs and APIs (metric tons), spending and price per kilogram (inflation-adjusted dollars), and market concentration measured by the Herfindahl-Hirschman Index (HHI). An HHI less than 1500 indicates an unconcentrated (ie, competitive) market, 1500 to 2500 indicates a moderate concentration, and greater than 2500 indicates high concentration.

**Results:**

The final sample included 50 FDF-originating countries and 52 API-originating countries. Compared with the annual volume of US antibiotic FDF imports in 1992, the annual volume in 2024 increased 2595.0%, while the annual volume of API imports remained relatively stable. Mean inflation-adjusted importation prices for FDFs decreased from $1836.03 per kg in 1992 to $177.44 per kg in 2024. For APIs, mean prices decreased from $351.74 per kg in 2003 to $65.69 per kilogram in 2024. From 2020 to 2024, India was the leading originating country for FDFs (31.9% of the total imported volume and 18.2% of the total imported cost), followed by Italy (13.4% of the total volume and 22.4% of the total cost). China was the leading originating country for APIs (62.6% of the total imported volume and 28.7% of the total cost), followed by Bulgaria (16.1% of the total volume and 3.8% of the total cost). Italy was the originating country for 2.6% of API imported volume but accounted for 27.9% of the importation costs. HHI revealed that FDF importation has become unconcentrated since 2020 (HHI, 1500-2500), while API importation markets are highly concentrated (2024 HHI, >5000).

**Conclusions and Relevance:**

This study found that US antibiotic importation relies on diversified global sources for FDFs but primarily on China for APIs. Policies to strengthen domestic production and diversify sourcing are critical to mitigate supply chain vulnerabilities. Improved traceability and targeted strategies for specific antibiotics are recommended to safeguard public health and national security.

## Key Points

**Question:**

Where do antibiotics for the US market come from?

**Findings:**

This cross-sectional study analyzed 1992 to 2024 antibiotic importation records and found 50 originating countries of finished dosage forms and 52 countries for active pharmaceutical ingredients as well as a significant increase in annual antibiotic imports and a decrease in importation prices. In the past 5 years, India was the leading originating country for finished dosage forms, with China as the leading originating country for antibiotic active pharmaceutical ingredients.

**Meaning:**

US relies on diversified global sources for antibiotic drugs but primarily on China for antibiotic active ingredients; policies to strengthen domestic production and diversify sourcing are critical to mitigate supply chain vulnerabilities.

## Teaser

This cross-sectional study evaluated the global sources of finished drug forms and active pharmaceutical ingredients for antibiotics imported into the US.

## Introduction

The US has faced persistent drug shortages in the past decade, with over 100 prescription drugs in short supply as of early 2025.^1^ Drug shortages compromise patient care and pose a threat to public health.^2^ Antibiotic shortages are particularly ominous. Antibiotics are a key part of routine medical care against bacterial infections and are often lifesaving, especially for critically ill patients.^3^ Antibiotics are also essential medical countermeasures against biothreat agents, such as anthrax, and are essential for supporting military operations in combat zones. Despite their importance, antibiotics are 42% more likely to experience shortages compared with most other types of drugs.^4^

There is a considerable lack of clarity regarding the sources of antibiotics in the US, primarily due to the difficulty in tracing their origins. Most antibiotics are unpatented generic drugs, which allows multiple global manufacturers to produce the same drug.^3,5,6^ Even when the manufacturer of a finished dosage form (FDF), ie, a finished drug product, is identified, the source of its active pharmaceutical ingredient (API) often remains uncertain.^6^ APIs—the substances that provide a drug’s therapeutic effect—are the most important components of generic drug production, largely determining the quality of the final product.^6^ Most generic FDF manufacturers outsource API production to third-party manufacturers, which can be located anywhere in the world.^6^

Growing concerns are emerging about the US overdependence on global manufacturers for its antibiotics supply.^7,8^ As domestic factories have closed or shifted production to other drugs, the US generic drug industry can no longer produce certain key antibiotics, such as penicillin and doxycycline.^7^ Intense competition from foreign products has critically contributed to this shift. In 2004, the last US manufacturing plant of penicillin APIs ceased operations due to price competition from foreign countries, leaving the domestic market entirely dependent on foreign-made penicillin.^9^ Reliance on foreign sources for pharmaceutical supply exposes the US to supply chain disruptions beyond its control, such as shipping delays, lockdowns, and export bans imposed by other countries.^10^ In addition, the US Food and Drug Administration’s ability to inspect overseas manufacturing plants may be limited, especially during crises, which can potentially compromise the quality and safety of available drugs.^10^

A particular concern is market concentration, ie, the extent to which supply chains are controlled by a small number of firms or countries.^11^ In highly concentrated markets, a few suppliers may substantially affect outcomes, either through restricting supply (resulting in higher prices) or through oversupplying (resulting in prices below profitable levels to discourage competition).^11^ Concerns are particularly acute when supply chain dependency involves nonpartner countries, such as China.^7,8^ Propelled by subsidies from the Chinese government, Chinese-made antibiotics can offer prices below the cost of production, helping drive US and other global manufacturers off the market.^9^ Nearly all US supplies of penicillin API are now sourced from China.^9,12^ Chinese-produced APIs are also used by other major global antibiotic manufacturers, such as those in India,^13,14^ obscuring the understanding of the US reliance on Chinese production.

Multiple attempts have been made to estimate the level of US dependency on foreign countries for its prescription drug supply. However, a key challenge is the difficulty in acquiring the necessary information. According to the US Senate Committee on Homeland Security and Governmental Affairs, neither the federal government nor the pharmaceutical industry have end-to-end visibility of the pharmaceutical supply chain, mainly due to the lack of data on the various stages of manufacturing, which include APIs and FDFs.^15^ Some studies have examined the number of manufacturers registered with the US Food and Drug Administration, a measure of manufacturing capability that may not accurately reflect production levels.^6,16,17^ Other studies have examined sales dollars, which may underestimate the percentage of the supply coming from countries that tend to supply cheaper products, such as China.^18,19^ To properly identify the origins of the US antibiotic supply, it is therefore necessary to examine sources by volume and separately for APIs and FDFs. This study examined importation records for FDFs and APIs over a 33-year period with the aim of identifying the global sources of the US antibiotic supply to inform policies to strengthen the US antibiotic supply, helping protect public health and enhance national security.

## Methods

According to the Common Rule, this study was not considered human participant research and was therefore exempt from review and the informed consent requirement. The study followed the Strengthening the Reporting of Observational Studies in Epidemiology (STROBE) reporting guidelines for observational studies.^20^

The data for this cross-sectional study were extracted from USA Trade Online, a platform that contains the complete list of US export and import records for all commodities collected by the US Customs and Border Protection from air, vessels, and overland ports.^21,22^ The platform tracks monthly US imports of all commodities; browsable commodity codes can be created for products with at least 3 US importers and an annual US import value of at least $1 million.^23^ Importation records for antibiotic FDFs and APIs were extracted over a 33-year period from January 1992 to December 2024. Antibiotics documented as primarily for veterinarian use were excluded. The commodity codes examined in this study are presented in eTable 1 in Supplement 1.

From each importation record, the originating country, the imported volume in metric tons, and the imported cost in US dollars were extracted. Importation costs were adjusted for inflation using the Consumer Price Index report for July 2024.^24^ Imported volume and cost were aggregated separately for FDFs and APIs across the 33 years for each originating country. Originating countries were excluded from the analysis if they appeared in the dataset for only 1 year or if the aggregated 33-year importation volume was less than 1 metric ton (1000 kg). Based on these criteria, some countries could be included in the FDF importation sample but were excluded from the API sample or vice versa (eTable 2 in Supplement 1).

### Statistical Analysis

Annual imported volumes of FDFs and APIs originating from India, China, other Asian countries (Bangladesh, Indonesia, Israel, Japan, Jordan, South Korea, Malaysia, Oman, Saudi Arabia, Singapore, Thailand, Turkey), European countries, the Americas, and other countries (Australia, Morocco, New Zealand, South Africa) were tracked over 33 years and the past 5 years (2020-2024). Importation volumes originating from each country are reported as a proportion of the total annual volume (eTable 3 in Supplement 1). Mean (SD) prices were calculated by dividing the total importation cost by the total importation volume in each year originating from each country. To measure market concentration for FDFs and APIs, the Herfindahl-Hirschman Index (HHI) was calculated using each country as a unit and annual importation volume as the share of the market.^25,26^ The HHI ranges from 0 (highly diversified) to 10 000 (single-source monopoly). Under US antitrust benchmarks, an HHI less than 1500 indicates an unconcentrated (competitive) market, 1500 to 2500 indicates moderate concentration, and more than 2500 is highly concentrated. This provides a framework to interpret how dependent the US is on any single country for antibiotics.^26^ Descriptive statistics and data visualizations were generated using RStudio, version 4.3.2 (Posit, PBC).

## Results

**Figure 1.**
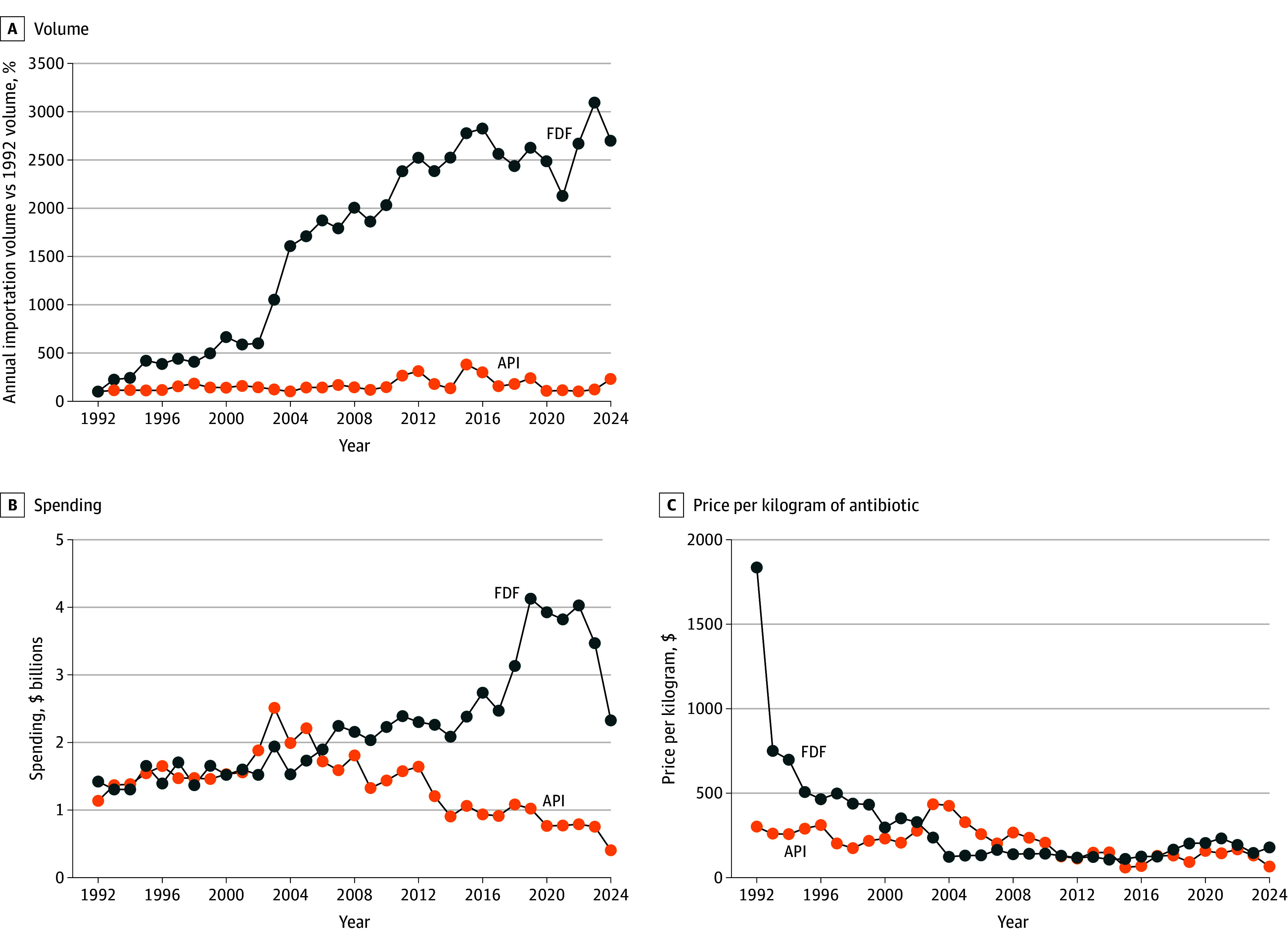
Trends in Annual Antibiotic Importation Volume, Spending, and Price, 1992 to 2024 A, The change in annual volume of antibiotic finished dosage form (FDF) and active pharmaceutical ingredient (API) imports over the study period compared against the 1992 annual volume. B and C, Annual spending and mean price per kilogram were adjusted for inflation using the Consumer Price Index report for July 2024.

US imports of antibiotic FDFs and APIs between 1992 and 2024 were analyzed. The final sample included 50 FDF-originating countries and 52 API-originating countries. The annual volume of US antibiotic FDF imports increased from 1992 to 2024 (2595.0% increase in 2024 vs 1992), while the annual API import volumes remained relatively stable over the same period (Figure 1A). Annual spending on antibiotic FDF imports increased from $1.5 billion in 1992 to $4.1 billion in 2019. Annual spending on API imports progressively declined, having remained at or below $1.0 billion since 2015 (Figure 1B). Inflation-adjusted average importation prices declined significantly for both FDFs and APIs from 1992 through 2024. FDF mean inflation-adjusted importation prices dropped from $1836.03 per kilogram in 1992 to $177.74 per kilogram in 2024. API mean importation prices declined from $351.74 per kilogram in 2003 to $65.69 per kilogram in 2024 (Figure 1C).

During the 33-year study period, a total of 50 different originating countries for FDFs and 52 different originating countries for antibiotic APIs were recorded, with 44 countries appeared in both FDF and API import records (eTable 3 in Supplement 1). Nine countries—Belgium, Canada, China, Germany, Ireland, Italy, Japan, Switzerland, and the United Kingdom—had records for both FDF and API importation in all 33 years. The number of different FDF-originating countries recorded annually increased from 21 in 1992 to 37 in 2024 (eFigure 1 in Supplement 1). For APIs, the number of originating countries increased from 32 in 1992 to a peak of 43 countries in 2008, declining to 34 by 2024 (eFigure 1 in Supplement 1).

**Figure 2.**
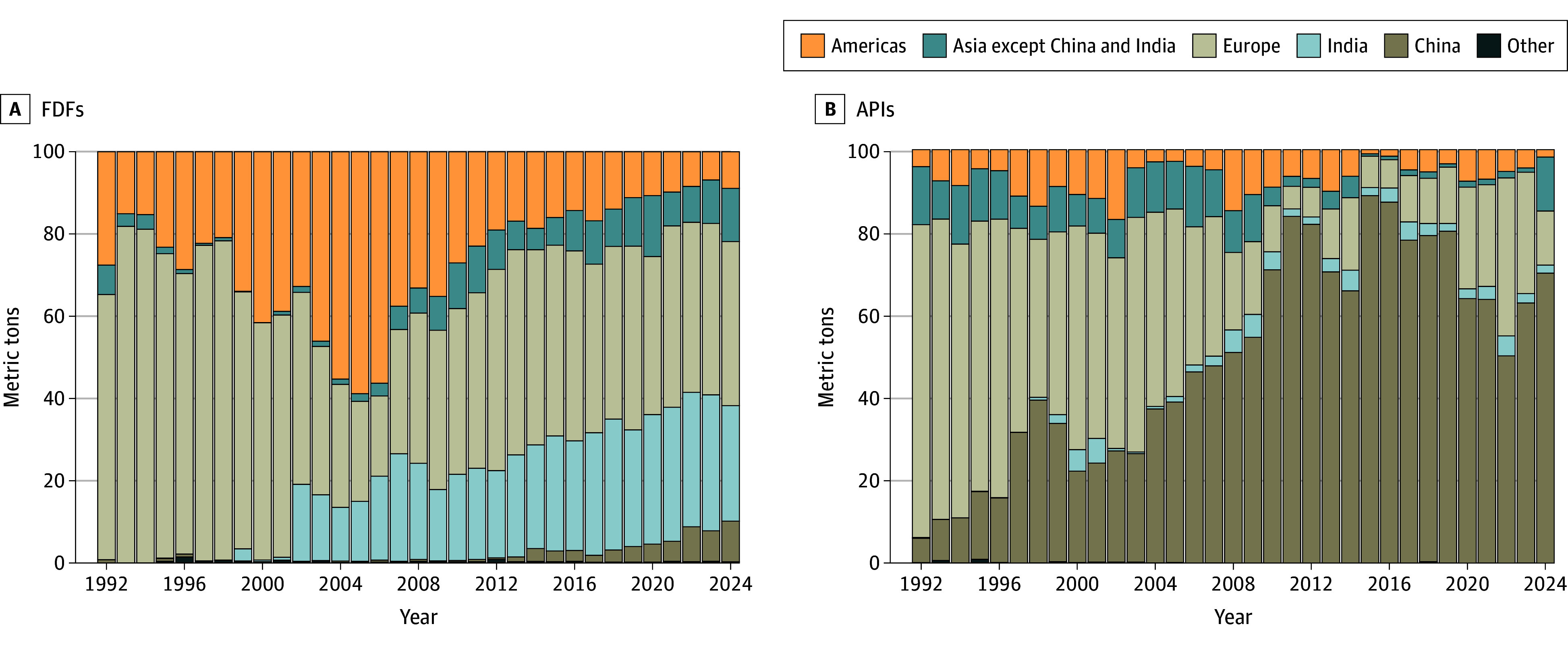
Annual Antibiotic Finished Dosage Form (FDF) and Active Pharmaceutical Ingredient (API) Importation Volume by Originating Country From 1992 to 2024 The full list of countries by region are provided in eTable 3 in Supplement 1. Countries in Asia except China and India include Bangladesh (FDF only), Indonesia (API only), Israel, Japan, Jordan, South Korea, Malaysia (API only), Oman (API only), Saudi Arabia, Singapore, Thailand (API only), and Turkey. Countries in Other include Australia, Morocco (FDF only), New Zealand, and South Africa.

Historically, antibiotic FDF imports originated predominantly from European countries. However, this dominance has decreased significantly over time (Figure 2A). In 1992, Europe accounted for 64.4% of FDF imports by volume, but by 2024, its share had dropped to 39.3%. India, which had no record of FDF imports until 2002, has emerged as a major supplier, contributing to 28.1% of imports in 2024. China and other Asian countries accounted for 9.9% and 12.9% of FDF imports in 2024. American countries (primarily Mexico and Canada), which led FDF imports in the mid-2000s and reached a peak share of 58.9% in 2005, declined to 8.9% by 2024.

Antibiotic API imports experienced a shift in sourcing over the study period (Figure 2B). In 1992, Europe supplied 75.7% of API imports, but by 2024, Asia dominated: China accounted for 70.1% of API imports, with India and other Asian countries contributing 1.9% and 13.1%, respectively (Figure 2B). Europe accounted for 13.1% of API imports in 2024 and North America accounted for 1.8%.

Based on aggregated importation volume from 2020 to 2024, India was the leading originating country for antibiotic FDFs (31.9% of the total imported volume and 18.2% of the total imported cost), followed by Italy (13.4% of the total imported volume and 22.4% of the total imported cost), Jordan (9.3% of the total imported volume and 1.4% of the total imported cost), Switzerland (8.5% of the total imported volume and 1.7% of the total imported cost), and Canada (7.7% of the total volume and 37.3% of the total cost) (Table). China was the leading originating country for APIs (62.6% of the total imported volume and 28.7% of the total imported cost), followed by Bulgaria (16.1% of the total imported volume and 3.8% of the total imported cost). Spain, Mexico, Israel, India, Italy, Croatia, and Canada followed with 3.2% to 0.9% of the total imported volume each. Although Italy was the originating country for 2.6% of the total imported volume of APIs, it accounted for 27.9% of the total importation costs. Among the top 15 originating countries for FDFs, 9 countries (60.0%) were also among the top 15 originating countries for APIs.

**Table.** Top 15 Originating Countries for US Antibiotic Importation, 2020 to 2024

Rank	Originating country	5-y Importation volume, metric tons, (%)^a^^,^^b^	5-y Importation cost, $ million, (%)^b^^,^^c^
**Finished dosage forms **			
Total	All	92 525 (100)	16 026 (100)
1	India	29 534 (31.9)	2911.87 (18.2)
2	Italy	12 358 (13.4)	3588.19 (22.4)
3	Jordan	8342 (9.0)	216.30 (1.4)
4	Switzerland	7841 (8.5)	272.52 (1.7)
5	Canada	7080 (7.7)	5972.20 (37.26)
6	China	6378 (6.9)	751.08 (4.7)
7	Austria	5530 (6.0)	349.25 (2.2)
8	Portugal	3287 (3.6)	164.92 (1.0)
9	Spain	3171 (3.4)	128.08 (0.8)
10	Romania	1658 (1.8)	37.16 (0.2)
11	Israel	1373 (1.5)	172.41 (1.1)
12	Croatia	1202 (1.3)	100.27 (0.6)
13	Slovenia	1047 (1.1)	54.49 (0.3)
14	Brazil	993 (1.1)	137.59 (0.9)
15	Hungary	527 (0.6)	34.61 (0.2)
**Active pharmaceutical ingredients **			
Total	All	26 745	3183
1	China	16 745 (62.6)	911.99 (28.7)
2	Bulgaria	4294 (16.1)	121.93 (3.8)
3	Spain	858 (3.2)	50.71 (1.6)
4	Mexico	826 (3.1)	40.88 (1.3)
5	Israel	796 (3.0)	21.44 (0.7)
6	India	762 (2.9)	144.20 (4.5)
7	Italy	579 (2.2)	888.65 (27.9)
8	Croatia	474 (1.8)	124.37 (3.9)
9	Canada	281 (1.1)	9.51 (0.3)
10	Brazil	263 (1.0)	55.24 (1.7)
11	Austria	203 (0.8)	66.82 (2.1)
12	Taiwan	107 (0.4)	52.06 (1.6)
13	Singapore	106 (0.4)	249.42 (7.8)
14	United Kingdom	82 (0.3)	16.29 (0.5)
15	Denmark	78 (0.3)	196.57 (6.2)

^a^ Volumes standardized to metric tons.

^b^ Percentages do not add up to 100% because only the top 15 countries are displayed.

^c^ Costs were adjusted for inflation using the Consumer Price Index report for July 2024.

**Figure 3.**
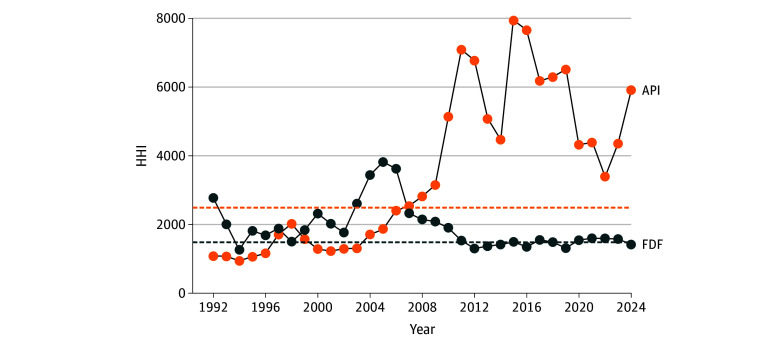
Market Concentration of Antibiotic Finished Dosage Form (FDF) and Active Pharmaceutical Ingredient (API) Imports According to the Herfindahl-Hirschman Index From 1992 to 2024 The Herfindahl-Hirschman Index (HHI) ranges from 0 to a maximum of 10 000 (ie, when a single firm concentrates 100% of the market). Generally, markets with an HHI lower than 1500 (blue dashed line) are considered unconcentrated (diversified); markets with HHI between 1500 and 2500 (orange dashed line) are considered moderately concentrated, and markets with an HHI higher than 2500 are considered highly concentrated.

FDF importation was moderately concentrated between 1992 and 2000 (HHI, 1500-2500) and it has become unconcentrated since 2020 (HHI, <1500), suggesting increasing competition (Figure 3). API importation has become significantly concentrated over time, with an HHI higher than the threshold of 2500 indicating a highly concentrated market since 2008 (2024 HHI, >5000). While China stands out as the key originating country for APIs, India, Canada, Italy, and others share the market for FDFs (eFigure 2 in Supplement 1).

## Discussion

Between 1992 and 2024, the US increased the annual importation of antibiotic FDFs from the global market by approximately 26-fold, while annual API importation volumes did not significantly change. Import prices decreased over 90% for FDFs and about 80% forAPIs but varied significantly across originating countries, often creating a disconnect between the proportion of the volume and the spending originating from each country. Countries with lower prices, such as India and China, accounted for a greater proportion of the import volume but a lower proportion of the spending, while countries with higher prices, such as Italy and Canada, accounted for a lower proportion of the import volume but a larger proportion of the spending.

Currently, the US imports antibiotic FDFs primarily from India, Canada, and European countries, representing a nonconcentrated market. This finding reflects decades of incentives for pharmaceutical production in these countries, such as the availability of public sector investment, private sector entrepreneurs, and a strong technological base.^14^ China dominated the imports of antibiotic APIs, with more than 60.0% of imported APIs from 2020 to 2024. This is likely the result of public subsidies as well as the lower costs of labor and lower environmental regulation.^27^ Because APIs must be transformed into FDFs to allow for clinical use, it can be assumed that imported APIs are being transformed into FDFs by US domestic manufacturers, unless imported APIs are being subsequently exported still in API form.

To the extent that domestic FDF manufacturers rely on imported APIs for their antibiotic production, the study’s findings suggest that the US domestic antibiotic FDF industry relies heavily on Chinese APIs. Interruptions in Chinese exports due to geopolitical tensions, trade wars, manufacturing quality issues, or others could therefore substantially impact US domestic antibiotic production. Yet, the scale of US domestic antibiotic production remains unclear. Quantifying volume and pricing trends in domestic antibiotic FDF and API production would provide critical information to better understand the foreign dependency of the US antibiotic supply chain.

Because previous research indicates that US antibiotic consumption has remained relatively stable over time,^28,29^ the marked growth in FDF imports identified in this study together with stable API importation levels suggest a trend toward increasing reliance on antibiotics made outside of the US rather than expanding the domestic FDF production capacity. The diversification of FDF importation markets identified in this study may be an underestimation of the US dependency on China if the countries exporting antibiotic FDFs to the US also rely on China for their API supply.^13,14,30^

The main benefit from the growth in US antibiotic importation has been a marked decrease in FDF and API importation prices. However, the extent to which lower antibiotic importation costs has translated into lower prices for American consumers remains unclear. Evidence suggests no substantial declines in the median US National Average Drug Acquisition Cost of antibiotics over time, or the opposite, with some formulations increasing prices by 90% or more.^31^

A downside of relying on the global market is that the US competes for supply with other countries, and the domestic industrial base may not readily compensate for sudden dips in supply. A recent investigation of global API production identified that each global generic API manufacturer supplied, on average, 2 other global markets in addition to the US.^6^ The consequences of global competition were acutely seen during the COVID-19 pandemic, when countries competed for resources while some manufacturer countries implemented export bans to protect their domestic pharmaceutical supply.^10^

Enhancing US antibiotic supply resilience requires evaluating the upstream supply chains used by FDF-originating countries, the scale and adequacy of US domestic antibiotic production, and how domestic production and prices compare with those of antibiotic imports. Research should examine upstream supply chains, including API sources used by FDF-originating countries, and monitor importation volumes instead of costs, because costs are influenced by prices.^32^ It is also important to examine the specific drugs imported from each country, as different prices may reflect differences in the types of products originating from each source. Risk factors that include market concentration metrics, like the approach developed by Nemoto et al^33^ using HHI in the context of Japan, can offer a helpful template. To identify high dependency on specific pharmaceutical ingredients, HHI greater than 2500 was used to define a high-risk flag, which was recommended as an early-warning metric for procurement agencies.

The patterns identified here are subject to change over time and influence by global and US domestic policies. Recent policy proposals have suggested imposing tariffs on products imported from countries, such as China and Canada.^34,35^ Applying such policies to FDFs and APIs would likely lead to higher prices, increasing financial burdens on the US health care system.^35^ Tariffs could also exacerbate drug shortages, particularly for essential medications. Although US buyers could diversify imports by turning to other emerging suppliers, the transition would likely take time and could introduce new risks, such as inadequate capacity to meet increased demand. Tariffs could also discourage countries’ further investment in pharmaceutical exports to the US, creating long-term supply chain vulnerability to disruptions.^36^

### Limitations

This study had several limitations. First, although the data used in this study are not expected to be subject to sampling error (because the data reflect the entirety of imports and exports recorded by Customs and Border Protection), the data could still be subject to nonsampling errors, such as reporting errors, undocumented shipments, low value estimations, and others.^22^ Second, antibiotic importation was analyzed in aggregate, without differentiating between drugs. It is possible that importation patterns of specific products differed from the overall trends observed. Future research should explore the supply chain dynamics of specific antibiotics, particularly those with high clinical value or limited substitutability. Third, the study did not differentiate between single-source branded drugs and multisource generic drugs, which often have distinct supply chains. Fourth, the data did not contain information on the US domestic antibiotic production, preventing a more thorough assessment of the US reliance on foreign sources for its antibiotic supply. Our assumption that imported antibiotics were intended for domestic consumption may not hold if the imported products were subsequently exported from the US to other international markets. Although we excluded products identified as primarily for veterinary use, we cannot exclude that some of the antibiotics imported for human consumption may be used in veterinary settings. Finally, by focusing solely on antibiotics, which have more stringent manufacturing requirements, lower profit margins, and higher vulnerability to market exit by producers than the average drug,^3,4,5^ the study results may not be generalizable to drugs in other therapeutic classes.

## Conclusions

Antibiotics are essential medicines for clinical practice, emergency preparedness, and national security, making it imperative to understand the origins of the antibiotic supply to safeguard, promote, and improve health in the US. To protect national security and enhance the US preparedness against shocks to global pharmaceutical supply, it is important to improve the traceability of antibiotic FDF and API sources. Expanding domestic manufacturing capacity (onshoring) and diversifying antibiotic supply chains through expanding sourcing from allied countries (friendshoring) are critical steps that depend on a detailed understanding of antibiotic supply chains and global sources. Policymakers should consider key FDF and API origin countries when imposing tariffs, revising trade agreements, or implementing manufacturing incentives to avoid supply disruptions. Specific drugs of critical interest should warrant targeted supply chain analysis and tailored strategies.

## Notes

Supplement 1. **eTable 1.** US Census Trade Commodity Codes Examined in the Study **eTable 2.** Inclusion and Exclusion Criteria for Originating Countries Examined in the Study **eTable 3.** US Antibiotic Importation Volume and Importation Cost by Originating Country, Aggregated 1992-2024 **eFigure 1.** Number of Originating Countries Recorded in US Antibiotic Importation Records, per Region and Year, 1992-2024 **eFigure 2.** Market Share of Selected Countries in US Antibiotic Importation, 1992-2024

Supplement 2.
Data Sharing Statement
