# Supplementary material for: US Antibiotic Importation and Supply Chain Vulnerabilities
Source: JAMA Health Forum. 2025 Oct 3;6(10):e253871. doi: 10.1001/jamahealthforum.2025.3871 (PMC12495497; doi:10.1001/jamahealthforum.2025.3871)
Supplement: Supplement 1. — eTable 1. US Census Trade Commodity Codes Examined in the Study eTable 2. Inclusion and Exclusion Criteria for Originating Countries Examined in the Study eTable 3. US Antibiotic Importation Volume and Importation Cost by Originating Country, Aggregated 1992-2024 eFigure 1. Number of Originating Countries Recorded in US Antibiotic Importation Records, per Region and Year, 1992-2024 eFigure 2. Market Share of Selected Countries in US Antibiotic Importation, 1992-2024 [file jamahealthforum-e253871-s001.pdf]

Supplemental Online Content

Socal MP, Sun Y, Ballreich JM, Lambert JD, Dai T, Dada M. US antibiotic importation and supply chain vulnerabilities. *JAMA Health Forum.* 2025;6(10): e253871.
doi:10.1001/jamahealthforum.2025.3871

eTable1. US Census Trade Commodity Codes Examined in the Study

eTable 2. Inclusion and Exclusion Criteria for Originating Countries Examined in the Study

eTable 3. US Antibiotic Importation Volume and Importation Cost by Originating Country, Aggregated 1992-2024

eFigure 1. Number of Originating Countries Recorded in U.S. Antibiotic Importation Records, per Region and Year, 1992-2024

eFigure 2. Market Share of Selected Countries in U.S. Antibiotic Importation, 1992-2024

This supplemental material has been provided by the authors to give readers additional information about their work.

© 2025 Socal MP, et al. *JAMA Health Forum*.

eTable1. US Census Trade Commodity Codes Examined in the Study

Pharmaceutical Products (Final Dosage Forms)[a]	
300310	Medicaments Containing Penicillins Or Derivatives
300320	Medicaments Containing Antibiotics Not Elsewhere Specified
300410	Penicillins Or Streptomycins & Derv, Dosage Form
	Note: 3004101010 excluded because indicates drugs for veterinarian use[b]
300420	Antibiotics Not Elsewhere Specified Or Included, In Dosage Form
	Note: 3004200010 excluded because indicates drugs for veterinarian use[b]

Organic Chemicals (Includes APIs, active pharmaceutical ingredients)[a]	
2941	Antibiotics

NOTES:

All data collected are from the catalogue scope of the 10- digit Harmonized Tariff Schedule of the United States (HTS) codes, extracted from the U.S. Census Trade Online platform for the period of January 1992 to July 2024. HTS codes are hierarchical. Thus, each 4- or 6-digit category listed above automatically includes all the 10-digit subcategories that it contains, except in the cases where a 10-digit subcategory is explicitly listed in the table as having been excluded due to its primarily veterinarian use.

[a]All importation volumes were standardized to kilograms and all importation costs were adjusted to 2024 values to account for inflation.

[b]Drugs for veterinarian use were excluded from the sample

© 2025 Socal MP, et al. *JAMA Health Forum*.

eTable 2. Inclusion and Exclusion Criteria for Originating Countries Examined in the Study

	**Finished Dosage Forms (FDFs)**	**Active Pharmaceutical Ingredients (APIs)**
N countries with U.S. imports 1992-2024	72	78
N countries excluded because only 1 year with importation	16	19
N countries excluded because total importation <1 metric ton	6	7
**Final N of originating countries included in the study**	50	52

NOTES:

Source: authors' analysis of U.S. Census Trade Online data, January 1992- July 2024. A total of 13 FDF-originating countries and 11 API-originating countries fulfilled both exclusion criteria.

© 2025 Socal MP, et al. *JAMA Health Forum*.

eTable 3. U.S. Antibiotic Importation Volume and Importation Cost by Originating Country, Aggregated 1992-2024

Panel A. Importation Volume and Cost by Originating Country for Antibiotic Finished Dosage Forms (FDFs), 1992-2024

Country	Region	Finished Dosage Forms (FDFs)		
		Years with imports	Total importation volume, in metric tons (rank)	Total importation cost, in US$ million (rank)
India	INDI	29	98,525 (1)	5,855.5 (2)
Canada	AMER	33	90,169 (2)	6,407.8 (1)
Austria	EURO	32	46,412 (3)	1,748.4 (6)
Italy	EURO	33	41,998 (4)	4,956.7 (3)
Jordan	ASIA	20	19,902 (5)	262.5 (19)
Switzerland	EURO	33	18,751 (6)	1,759.1 (5)
United Kingdom	EURO	33	11,422 (7)	4,700.6 (4)
China	CHIN	33	10,569 (8)	507.0 (11)
Slovenia	EURO	24	10,411 (9)	345.9 (16)
Portugal	EURO	29	9,859 (10)	393.8 (15)
Israel	ASIA	28	8,798 (11)	1,074.4 (8)
Belgium	EURO	33	6,676 (12)	531.1 (10)
Croatia	EURO	19	6,020 (13)	423.9 (14)
Spain	EURO	29	6,019 (14)	195.8 (22)
Ireland	EURO	33	5,864 (15)	431.1 (13)
Germany	EURO	33	4,632 (16)	1,191.5 (7)
Norway	EURO	18	3,101 (17)	39.6 (26)
Brazil	AMER	26	3,038 (18)	284.3 (17)
Romania	EURO	18	2,121 (19)	41.2 (25)
Korea, South	ASIA	25	1,336 (20)	20.1 (29)
Denmark	EURO	20	1,279 (21)	115.4 (23)
Australia	OTHE	33	1,233 (22)	471.1 (12)
Mexico	AMER	32	1,109 (23)	11.6 (31)
Japan	ASIA	33	1,074 (24)	532.9 (9)
Hungary	EURO	19	747 (25)	31.0 (27)
France	EURO	31	552 (26)	242.9 (21)
Taiwan	CHIN	17	533 (27)	83.8 (24)
Netherlands	EURO	32	461 (28)	23.8 (28)

© 2025 Socal MP, et al. *JAMA Health Forum*.

Saudi Arabia	ASIA	9	199.1 (29)	9.9 (33)
Turkey	ASIA	10	184 (30)	10.0 (32)
South Africa	OTHE	5	122 (31)	265.3 (18)
Sweden	EURO	27	106 (32)	246.3 (20)
Greece	EURO	10	73 (33)	2.7 (38)
Macau	CHIN	17	68 (34)	16.5 (30)
Slovakia	EURO	13	61 (35)	8.3 (34)
New Zealand	OTHE	7	36 (36)	6.0 (36)
Bulgaria	EURO	6	18 (37)	2.2 (39)
Argentina	AMER	6	16 (38)	<1
Morocco	OTHE	3	15 (39)	7.6 (35)
Dominican Republic	AMER	4	8.2 (40)	3.1 (37)
Ukraine	EURO	4	7.3 (41)	<1
Singapore	ASIA	11	5.5 (42)	<1
Bangladesh	ASIA	3	5.2 (43)	<1
Netherlands Antilles (through Apr 2011)	EURO	3	3.5 (44)	<1
Cyprus	EURO	5	2.7 (45)	<1
Malta	EURO	3	2.4 (46)	1.5 (40)
Hong Kong	CHIN	8	1.8 (47)	<1
Czech Republic	EURO	7	1.7 (48)	<1
Panama	AMER	5	1.6 (49)	<1
Finland	EURO	9	1.6 (50)	<1

Panel B. Importation Volume and Cost by Originating Country for Antibiotic Active Pharmaceutical Ingredients (APIs), 1992-2024

Country	Region	Active Pharmaceutical Ingredients (APIs)		
		Years with imports	Total importation volume, in metric tons (rank)	Total importation cost, in US$ million (rank)
China	CHIN	33	138,204 (1)	2665 (3)
Italy	EURO	33	18,260 (2)	4258.2 (1)
Mexico	AMER	33	12,710 (3)	265.3 (9)
United Kingdom	EURO	33	11,113 (4)	1390.1 (5)
Bulgaria	EURO	33	7,900 (5)	137.2 (11)
Austria	EURO	33	6,943 (6)	771.1 (4)
Singapore	ASIA	33	6,657 (7)	703.3 (8)
India	INDI	33	5,856 (8)	730.7 (6)

© 2025 Socal MP, et al. *JAMA Health Forum*.

Japan	ASIA	33	4,015 (9)	2676.5 (2)
Spain	EURO	33	3,263 (10)	330.4 (11)
Slovakia	EURO	27	2,989 (11)	29.5 (15)
Croatia	EURO	33	2,829 (12)	616.1 (9)
Netherlands	EURO	33	2,780 (13)	1689 (4)
Belgium	EURO	33	2,700 (14)	85.7 (17)
Germany	EURO	33	2,455 (15)	131.2 (12)
Brazil	AMER	31	2,354 (16)	153 (15)
Korea, South	ASIA	32	1,821.9 (17)	182.3 (14)
Portugal	EURO	33	1,684 (18)	372.8 (10)
Slovenia	EURO	33	1,478 (19)	287.3 (8)
Canada	AMER	33	1,285 (20)	39.9 (14)
Israel	ASIA	33	1,101 (21)	152.3 (10)
Ireland	EURO	33	1,082 (22)	293.2 (7)
Sweden	EURO	19	1,073 (23)	68.1 (19)
Denmark	EURO	33	1,038 (24)	737.6 (5)
Macau	CHIN	33	755 (25)	123.2 (16)
Hungary	EURO	33	674 (26)	263.4 (12)
Switzerland	EURO	33	647 (27)	1073.7 (6)
France	EURO	33	499 (28)	961.7 (7)
Norway	EURO	27	364 (29)	211.3 (13)
Saudi Arabia	ASIA	7	244 (30)	12 (23)
Hong Kong	CHIN	33	219 (31)	22.3 (21)
Australia	OTHE	30	207.1 (32)	44.6 (13)
Taiwan	CHIN	26	164 (33)	71.1 (18)
Jordan	ASIA	8	145.6 (34)	5.4 (25)
Romania	EURO	27	128 (35)	24 (20)
Czech Republic	EURO	32	107 (36)	9 (16)
Malaysia	ASIA	13	67.2 (37)	8.2 (17)
Netherlands Antilles (through Apr 2011)	EURO	3	26 (38)	7.4 (18)
Poland	EURO	13	17 (39)	1.3 (27)
Panama	AMER	4	16.6 (40)	<1
South Africa	OTHE	5	10.3 (41)	<1
Turkey	ASIA	12	9.9 (42)	5.7 (24)
Ukraine	EURO	14	8.5 (43)	3.9 (26)
Cyprus	EURO	2	8.2 (44)	<1
Colombia	AMER	4	7.8 (45)	<1
Oman	ASIA	9	7.6 (46)	<1
Malta	EURO	8	5.6 (47)	15.1 (22)
Uruguay	AMER	5	4.6 (48)	<1
Thailand	ASIA	5	3.1 (49)	<1
Indonesia	ASIA	4	1.8 (50)	<1
New Zealand	OTHE	5	1.5 (51)	1.2 (19)
Iceland	EURO	3	1.2 (52)	<1

© 2025 Socal MP, et al. *JAMA Health Forum*.

Source: authors' analysis of U.S. Census Trade Online data, January 1992- July 2024. For a list of exclusion and inclusion criteria for commodities and originating countries, please see eTable1 and eTable 2 respectively. All importation volumes were standardized to kilograms and all importation costs were adjusted to 2024 values to account for inflation. Abbreviations: AMER - The Americas; ASIA: Asian Countries except China and India; CHIN: China (include Hong Kong, Macau and Taiwan); EURO: Europe; INDI: India; OTHE: Other countries besides the above five regions.

© 2025 Socal MP, et al. *JAMA Health Forum*.

eFigure 1. Number of Originating Countries Recorded in U.S. Antibiotic Importation Records, per Region and Year, 1992-2024

Panel A. Number of Originating Countries Recorded in U.S. Antibiotic Importation Records of Finished Dosage Forms (FDFs) per Region and Year, 1992-2024

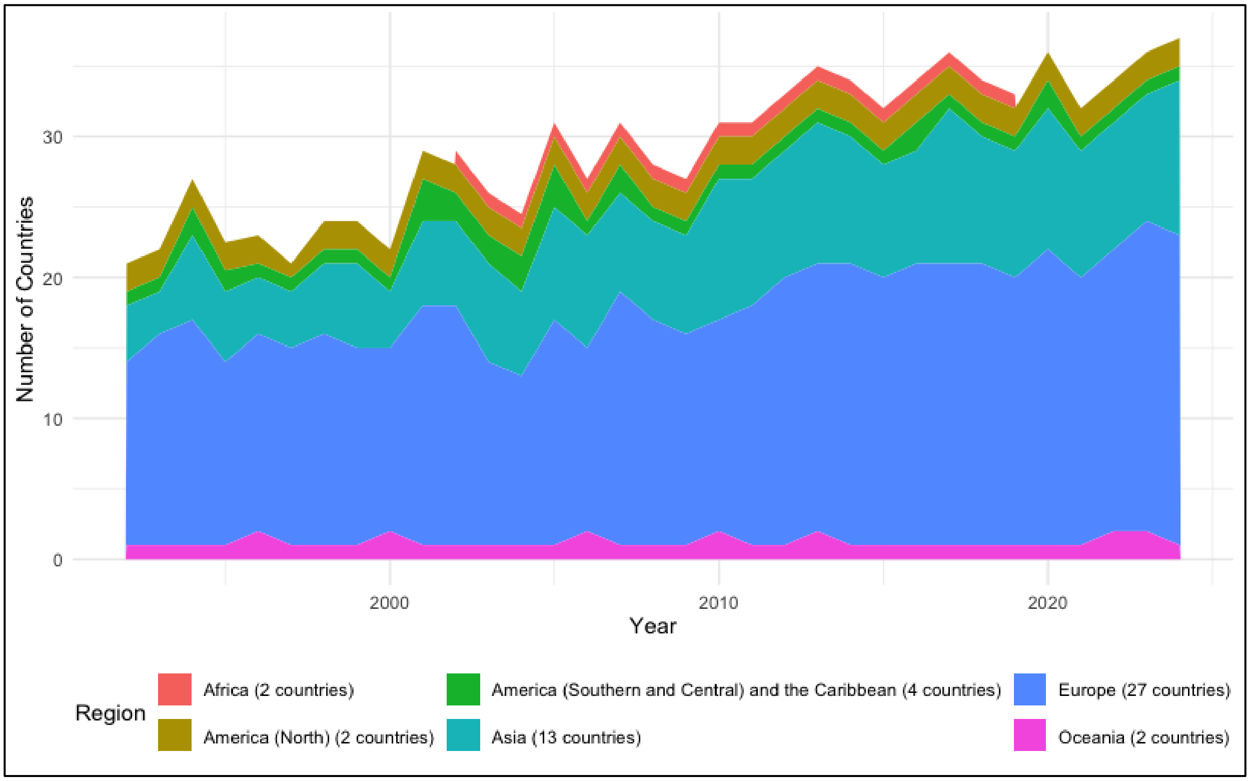

© 2025 Socal MP, et al. *JAMA Health Forum*.

Panel B. Number of Originating Countries Recorded in U.S. Antibiotic Importation Records of Active Pharmaceutical Ingredients (APIs) per Region and Year, 1992-2024

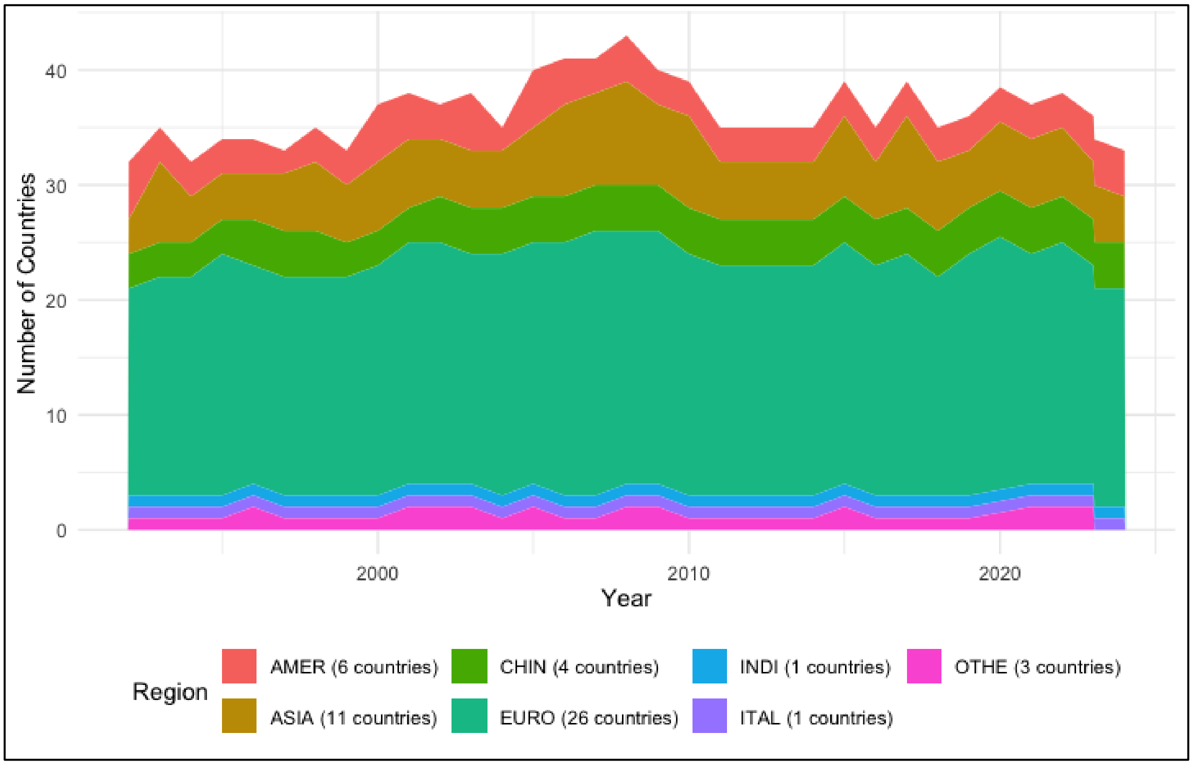

Source: authors' analysis of U.S. Census Trade Online data, January 1992- July 2024. For a list of exclusion and inclusion criteria for commodities and originating countries, please see eTable1 and eTable 2 respectively. The counts of countries per region shown in the figure labels represent the number of different countries in each region that contributed to U.S. imports at any point in the study period.

© 2025 Socal MP, et al. *JAMA Health Forum*.

eFigure 2. Market Share of Selected Countries in U.S. Antibiotic Importation, 1992-2024

Panel A. Finished Dosage Forms (FDFs)

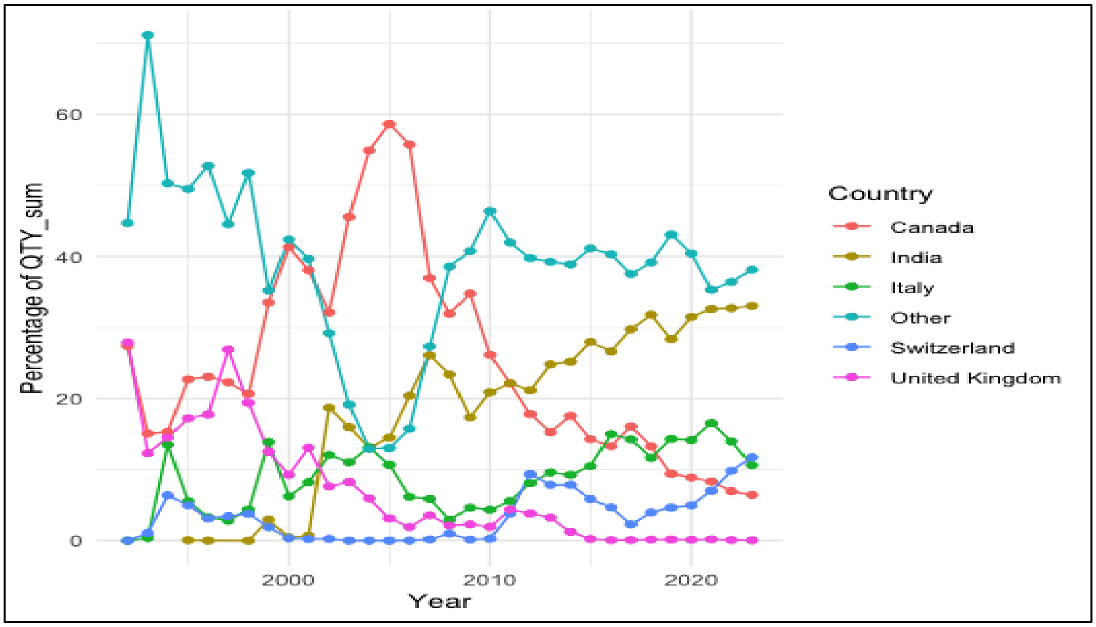

Panel B. Active Pharmaceutical Ingredients (APIs)

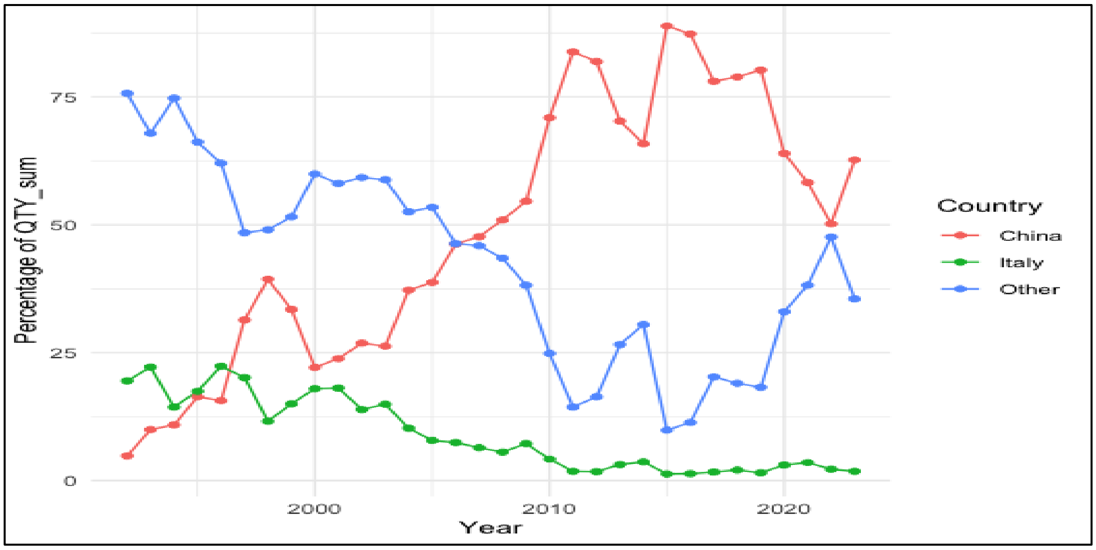

© 2025 Socal MP, et al. *JAMA Health Forum*.
