# Supplementary material for: US Antibiotic Importation and Supply Chain Vulnerabilities
Source: JAMA Health Forum. 2025 Oct 3;6(10):e253871. doi: 10.1001/jamahealthforum.2025.3871 (PMC12495497; doi:10.1001/jamahealthforum.2025.3871)
Supplement: Supplement 2. — Data Sharing Statement [file jamahealthforum-e253871-s002.pdf]

Data Sharing Statement

Socal. US Antibiotic Importation and Supply Chain Vulnerabilities. *JAMA Health Forum*. Published October 03, 2025. doi:10.1001/jamahealthforum.2025.3871

Data
Data available: Yes
Data types: Data (not involving human participants)
How to access data: https://usatrade.census.gov/
When available: With publication

Supporting Documents
Document types: None

Additional Information
Who can access the data: The database is maintained by the US Census Bureau and is available to everyone who registers in the platform
Types of analyses: for any purpose
Mechanisms of data availability: upon registration in the platform
Any additional restrictions: I am unaware of any restrictions.
